# The Effect of *Vitis vinifera *L. Leaves Extract on *Leishmania infantum*

**Published:** 2013

**Authors:** Rym Mansour, Najoua Haouas, Amel Ben Kahla-Nakbi, Saoussen Hammami, Zine Mighri, Farouk Mhenni, Hamouda Babba

**Affiliations:** a *Unité of Recherche 12-04, Chimie Appliquée and Environnement, Faculté of Sciences of Monastir, Monastir 5000, Tunisie.*; b *Laboratoire of Parasitologie-Mycologie (99UR/08-05),Faculté de Pharmacie, Département of Biologie, Clinique B, 1 Rue Avicenne, Monastir 5000,Tunisie.*; c *Laboratoire of Biodiversité and Biotechnologie Marine, Institut National des Sciences and Technologies of la Mer, Annexe Monastir, BP 59, Monastir 5000, Tunisie*

**Keywords:** *Vitis vinifera *L., Extraction, Leishmaniasis, Antileishmanial activity, Anthocyanins

## Abstract

*Vitis vinifera *L.is a traditional Asian herb widely used for different health problems. In the present research, the ethanolic and the aqueous extracts of *Vitis vinifera *L. leaves collected from shrub, grown in Tunisia, were prepared and evaluated for the antileishmanial activity against *Leishmani ainfantum *promastigotes. The inhibitory concentration 50 (IC_50_) was determined and the results showed that the etahnolic extract is more active than the aqueous one (IC_50_= 0.108 mg/mL). Microscopic observations showed that the ethanolic extract promoted the destruction of cytoplasmic and nuclear membranes of *Leishmani ainfantum *promastigotes and altered the overall shape of the cell. In order to explain the difference of antileishmanial activity between ethanolic and aqueous extracts, anthocyanins amount was determined by spectrophotometry. It was found that the ethanolic extract is richer in anthocyanins than the aqueous one which can explain the higher antileishmanial activity of the ethanolic extract.

## Introduction

Leishmaniasis is a real public health problem encountered in several countries over the world including Tunisia. In Tunisia, incidence was assessed to bemore than 4000 and 150 case per year for the cutaneous and the visceral leishmaniasis form respectively, (1, 2). Visceral leishmaniasis form is caused by *Leishmania *(*L.*) *infantum *species and is endemic in the North and the Center of Tunisia (3, 1). The same species may also be isolated in several cases of cutaneous leishmaniasis encountered in the same foci (4, 5).

 In absence of vaccination against the parasite *Leishmania *(*L*), the use of drugs remains the only way for treatment. In fact, pentavalentantimony such us Glucanthime^®^ (meglumine antimony) and Pentostam^®^ (sodium stibogluconate) were used for the treatment of both cutaneous and visceral leishmaniasis (6). In case of resistance, these drugs were substituted by amphotericin B and miltefosine^®^ (7). However, many signs of stibio-intoxication such as cardiac conduction disorders, hepatic cytolysis, dysfunction of renal tubular and pancreatitis have been attributed to these drugs (8, 9). In addition, resistance to pentavalent antimony has been reported in Bihar, India in 60% of treated patients (10, 11). Treatment failure was also observed in the Mediterranean region (12, 2). Accordingly, an urgent need to find new antileishmanial agents,more efficient and less harmful for patients is highly recommended.

*Vitis*(*V.*) *vinifera *(*Vitales, Vitaceae*), is an Asian native perennial woody vine. From different parts of this plant essentially fruits, several preparations used in folk medicine have been derived (13). In Ethnopharmacology, the infusion of the leaves of red varieties has been used as haemostatic and for diarrhea treatment. Fresh leaves have been used externally to heal wounds and to lance abscesses (14). Grape leaf-based medicines are traditionally used for diarrhea, hepatitis and stomachaches (15, 16, 13, 17). Grapes, seeds, and leaves have been used for preventing heart and blood vessels diseases, varicose veins, hemorrhoids, “hardening of the arteries” (atherosclerosis), high blood pressure, swelling after injury or surgery, heart attack and stroke. 

Moreover, grape leaf has been used for attention deficit-hyperactivity disorder (ADHD), chronic fatiguesyndrome (CFS), diarrhea, heavy menstrual bleeding, uterine bleeding, andcanker sores. It has been also used as a mild laxative forconstipation (16-24). 

Few studies have been conducted on the biological effects of leaves. Nilüfer *et al *have shown that the aqueous extract from leaves of *Vitis* *vinifera *L. possessantidiabetic and antioxidant activities (25). It was also mentioned that the aqueous extract of *Vitis vinifera *L. leaves shows antibacterial activity against *Escherichia coli,* *Enterococcus feacalis, Staphylococcus aureus* and *Vibrio alginolyticus *(26).The aim of this study wasto evaluate the antileishmanial activity of the aqueous and ethanolic extract of *Vitis vinifera *L*.* leaves and to quantify their anthocyanins amount as anthocyanins family is well known by the richness of its biological activities (27).

## Experimental

*Plant material*

The *Vitisvinifera*L*. *Black Grenache leaves are collected from “Bir Bou Ragueba”, a suburb in the city of Nabeul, Tunisia. Bir Bou Ragueba’s latitude is 10°25’ and its longitude is 36° 37 W. Leaves were collected on December 8^th ^2010. They were dried and reduced to fine powder (28).

*Preparation of extracts*

One gram of dried leaves of *Vitis vinifera L. *wasextracted with 20 mL of distilled water during 1 hour at 95°C. The mixture waspassed through a filter paper in order to remove plant debris. The aqueous extract was then sterilized by filtration through a 0.22 μm membrane filter. It was used freshly for the preparation of different concentrations in order to evaluate the antileishmanial activity. As for the ethanol extract, it was obtained by soxhlet with acidified ethanol (ethanol: HCl 99: 1 v / v: = 0.1 N HCl) till the reflux of this mixture. (29)

*Preparation of stock solution*

The aqueous extract was directly used. However, the ethanolic extract of *Vitisvinifera* L. leaves was initially dissolved in dimethyl sulfoxide (DMSO) at 1%. 10 mg of the ethanolicextract were dissolved in 100 μL of pure DMSO and then added to 900 μL of culture medium suitable for a final concentration of 10 mg/mL. This solution was sterilized by passage through a filter of 0.22 μm in a laminar flow hood. It was then diluted to different concentrations for the antileishmanial activity that the highest concentration tested is 1 mg/mL. In this way the final concentration of DMSO never exceeded 1% in the medium tested. This concentration had no effect on the growth of *Leishmania*. 

*Maintenance and counting of parasite*

The *L. infantum*strain (MHOM/TN/2010/44M) was isolated from a visceral leishmaniasis human case and typed by isoenzyme and molecular methods in the laboratory of Parasitology, Faculty of Pharmacy of Monastir, Tunisia. Promastigote Culture was maintained at 25 °C in RPMI 1640 medium supplemented with 10% of decomplemented fetal calf serum (Gibco Invitrogen Corporation, New York, NY) pH 7.0, in a cell culture dishes with weekly subcultures. The promastigotes were counted using Neubauer chamber and re-suspended in fresh medium at a final concentration of 1.0 × 10^6^ live promastigotes/ mL. The viability of *Leishmania *was assessed by mobility and lack of color in the presence of trypan blue.

*Anti-leishmanial activity*

The trials were conducted in a liquid medium in miroplaques of 96 round bottom wells. Promastigotes of the logarithmic phase were resuspended to a concentration of 10^6^*Leishmania*/

mL and were treated with ethanolic and aqueous extracts of *V. vinifera *L. leaves. For the ethanolic extract, the tested concentrations were 1, 10, 100, and 1000 μg/mL. While for the aqueous extract concentrations were 50 mg/mL, 25 mg/mL, 12.5 mg/mL and 6.25 mg/ mL. Two wells containing the negative control cultures without extract supplemented or not with 1% DMSO were used. A positive control well containing culture with Glucantime^®^, was also tested. The plates were incubated at 27 °C for 72 h to assess the anti-proliferative effect of the extracts. The number of mobile and viable promastigotes was quantified by counting the parasite using the Neubauer chamber. Inhibitory concentration 50 (IC_50_) was determined by the method of logarithmic regression analysis of data obtained. 

*Leishmania *cell morphology was also evaluated by deposing 20 μL of culture treated with ethanolic *V. vinifera *L. leaves extract on microscopic slides. After spreading and drying in ambient air, slides are fixed with absolute methanol, stained with 10% Giemsa, and examined under an oil immersion objective of the light microscope.

*Anthocyanins quantification*

The amount of anthocyanins in the extracts was determined according to the method of Giusti and Wrolstad. By changing the pH different values of absorbance wereobtained. Extracts were diluted 10 times in two buffers. The aqueous solution consistedof potassium chloride (0.025 M) with pH 1 and a second aqueous solution consisting of sodium acetate (0.045 M) after 15 min incubation at room temperature, the absorbance of both extracts weremeasured at 520 nm and 700 nm. The total anthocyanins content wasgiven in mg of cyanidin-3-glucoside (cy-3-glu) / liter (2), andthe equipment used wasan UV-visible CE- 202 spectrophotometer ( 27).

A = (A520 nm – A 700 nm) pH 1.0 - (A520 nm - A700 nm) pH 4.5 (1)

The concentration of anthocyaninswascalculated using this equation:

$C=\frac{A\times \mathrm{MW}\times \mathrm{DF}\times 10^{3}}{Ɛ\times ƪ}$                    (2)

**Figure 1:**
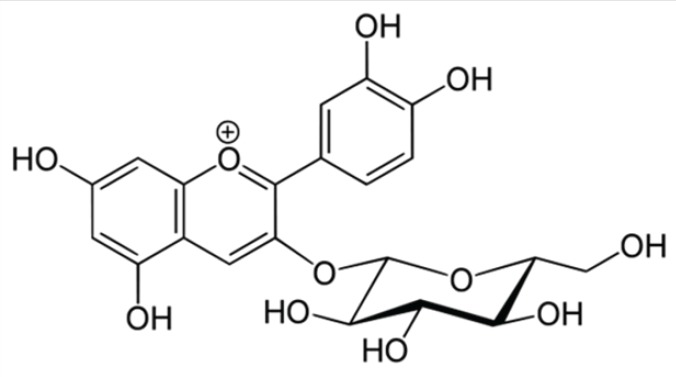
Chemical structure of cynidin-3-glucoside, Anti-leishmanialactivity of *Vitis vinifera*L. (*Vitales, Vitaceae*) leavesextracts, Rym Mansour

MW (molecular weight) = 449.2 g / mol for cyanidin-3-glucoside (Cyd-3-glu) (Figure 1);

DF = dilution factor

l=1 cm (cuvette width)

ε = 26 900 molar coefficient of extinction

in L^-1^xcm xmol^-1^, for Cyd-3-glu, and 10^3^ = conversion factor from g to mg.

*Statistical Analysis*

In order to assure the reproducibility of results, all experiments wererepeated thrice. The means and standard deviation were determined. The data werethen analyzed by SPSS version 17.0. The Student t-test was applied and the p-value less than 0.05 wasconsidered significant.

## Results

Vine leaves extracts were tested to evaluate their activity against promastigotes of *L.* *infantum*. For aqueous extract, IC_50_= 12.55 mg/ mL and for ethanolic extract IC_50_=108.85 μg/ mL. In addition, the ethanol extract showed an IC_50 _lower than glucanthime(IC_50_ = 8.504 mg/ ml). (Table1) 

In order to assess the viability of promastigotes of *L. infantum *in the presence of the ethanol extract, treated cultures were stained with May-Grunwald Giemsa and examined under an optical microscope.

**Table 1:** IC_50_ extracts of *Vitisvinifera*L. leaves against *L.infantum*

	**Aqueous extract**				**Ethanolic extract**				**Glucanthime**			
Concentrations (mg/L)	50	25	12.5	6.25	1	0.1	0.010	0.001	200	100	50	25
Percentage of Viability	0	3.	99.9	100	31.65	46.23	71.69	99.91	10.10	42.29	52.17	81.23
IC50 (mg/L)	12.53				0.108				8.504			

**Figure 2:**
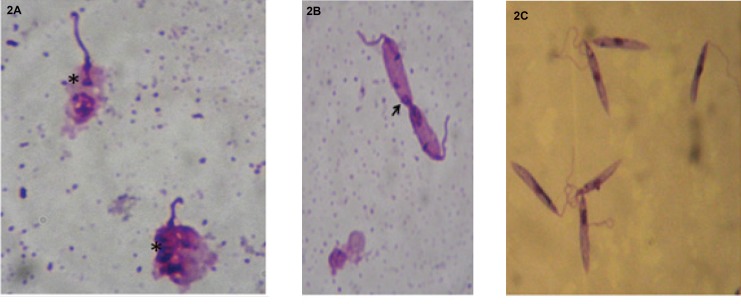
a: membrane destruction, Anti-leishmanialactivity of *Vitisvinifera *L. (*Vitales, Vitaceae*) leavesextracts, Rym Mansour. b: Unequal division, Anti-leishmanialactivity of *Vitis vinifera*L. (*Vitales, Vitaceae*) leavesextracts, Rym Mansour. c: Untreated*Leishmania* *infantum*culture., Rym Mansour

 The effects of ethanolic extract on *Leishmania *were different from those

caused by Glucanthime. Indeed, *Leishmania *incubated in the presence of the ethanolic extract of *V. vinifera *L. leaves showed the following result: destruction of cytoplasmic and nuclear membranes and, thus, altered the overall shape of the cell (Figure 2a, 2b). An abnormality at the cell division: an imbalance in the nuclear division (Figure 2b) and the division of the nucleus and the flagellum however,without duplication of the kinetoplast (Figure 2c).

 The quantity of anthocyanins present in the ethanolic and aqueous extracts was calculated using the spectrophotometric method according to the protocol of Giusti and Wrolstad. Thus, we found that the aqueous extract and ethanolic extract contained respectively 3.89 mg/g and 5.01 mg/g (mg anthocyanins/g of dry material). The ethanol extract wasricher inanthocyanins than the aqueous extract.

## Discussion

This study examined the antileishmanial activity of aqueous and ethanolic *V. vinifera* L. leaves extracts. The ethanolic extract showed a prominent activity against *L.* *infantum*promastigotes. *Vitisvinifera*leaves are rich in tannins, flavonoids, procyanidins and also contain organic acids, lipids, enzymes and vitamins (13, 30, 31, 17). Furthermore, the quantitative analysis of compounds found in leaves has also been evaluated by Monagas *et al. *(2006). They found that *V. vinifera *is rich in anthocyanins and other flavonoids (32).

We found that the difference between the IC_50_ could be the result of the difference in the anthocyanin content. The ethanol extract, having a higher content of anthocyanins than the aqueous extract, seems to be more effective against *L.* *infantum *promastigotes. This couldbe due to the number of hydroxyl groups of anthocyanins which is supposed to be the cause of the relative toxicity to microorganisms (33). 

Anthocyanins are the anthocyanidins in their glycoside form (linked to a sugar moiety). The anthocyanidins are composed of an aromatic ring attached to a heterocycle that contains oxygen, which is also linked by a carbon-carbon to a third aromatic ring (34). The anthocynidins are flavonoids which are, in their turn, a subdivision from the family of polyphenols. 

Anthocyanins display a wide range of biological activities including antioxidant, anti-inflammatory, antimicrobial and anticarcinogenic activities. In addition, they display a variety of effects on blood vessels, platelets and lipoproteins able to reduce the risk of coronary heart diseases (34). 

Kolodziej *et al. *(2001), have reported that proanthocyanidins possess antileishmanial activity against the species *L. donovari *with IC_50_ between 0.8 and 10.6 nM (35).

 Lewin *et al., *(2011) have also studied the antileishmanial activity of the flavonoids against *L. donovani *(36). Moreover, in 2010 Nour *et* *al*., found that *Ageratum conyzoides *methylated flavonoids have an antileishmanial activity against *L. donovani *with IC_50 _= 3.4 μg/mL (37). Flavonoids from *Agaricusblazei Murill* had an antileishmanial activity against *L.* *amazonensis*, *L. chagasi *and *L. major *(38). Several studies have shown that many natural compounds such as naphthoquinones, alkaloids, lignans and triterpenes possess antileishmanial

activity against many *Leishmania *species (39). Nevertheless, to our knowledge, our study is the first to show the correlation between anthocyanins content and IC50 of aqueous and ethanolic extracts of *V. vinifera L*. leaves against *L. infantum*promastigotes.

## Conclusion

For 50 years, pentavalent antimony was the drug most used for the treatment of leishmaniasis. These molecules have been used to treat all forms of leishmaniasis. However, recently liposomal amphotericin B has replaced the pentavalent antimony as a treatment of choice for visceral leishmaniasis. Nevertheless, these drugs have dangerous side effects. In this research, we have tried to evaluate the anti-leishmanial activity of aqueous and ethanolic extracts of *V. vinifera* L. leaves. Ethanolic extract showed interesting activity against *L. infantum *promastigotes. This extract is currently undergoing detailed investigations with the objective of isolating biologically active molecule(s). Based on the results of this study, we plan to develop bandages with *V. vinifera *extract inhibiting the dermatological proliferation of leishmaniasis, by nano-microencapsulation.

## References

[1] Aoun K, Jeddi F, Amri F, Ghrab J, Bouratbine A (2009). Actualités épidémiologiques de la leishmaniose viscérale en Tunisie. Méd. Mal. Infect.

[2] Faraut-GambarelliF, Piarroux F, Giusiano B, Marty P, Michel G, Faugère B, Dumon H (1997). in- vitro and in-vivo resistance of Leishmaniainfantumto meglumineantimoniate: a study of 37 strains collected from patients with visceral leishmaniasis. Antimicrob Agents Chemother.

[3] Carrio J, Riera C, Ga´ llego M, Portu´ s M (2001). Invitro activity of pentavalent antimony derivatives on promastigotes and intracellular amastigotes of Leishmaniainfantum strains from humans and dogs in Spain. Acta. Trop.

[4] Belhadj S, Pratlong F, Hammami M, Kallel K, Dedet J, Chaker E (2003). Human cutaneous leishmaniasis due to Leishmania infantum in the Sidi Bourouis focus (Northern Tunisia): epidemiological study and isoenzymatic characterization of the parasites. Acta. Trop.

[5] Kallel K, Pratlong F, Belhadj M, Cherif F, Hammami M, Dedet J, Chaker E (2005). La leishmaniose cutanée due à Leishmania infantum MON-24 en Tunisie : extension du foyer vers le centre du pays. Ann Trop. Med. Parasitol.

[6] Gradonil L, Gramiccia M, Pettoello M, Di Martino L, Nocerino A (1987). A new Leishmaniainfantumenzymatic variant, agent of an urban visceral case unresponsive to drugs. Trans. R. Soc. Trop. Med. Hyg.

[7] Gradonil L, Bryceson A (2001). The increase of risk factors for leishmaniasis worldwide. Trans. R. Soc. Trop. Med. Hyg.

[8] Lawn S, Aarmstrong M, Chilton D, Whitty C (2006). Electrocardiographic and biochemical adverse effects of sodium stibogluconate during treatment of cutaneous andmucosalleishmaniasis among returned travellers. Trans. R. Soc. Trop. Med. Hyg.

[9] Domingo P, Ferrer S, Kolle L (1996). Acute pancreatitis associated with sodium stibogluconate treatment in a patient with Human Immunodeficiency Virus. Arch. Intern. Med.

[10] Sundar S (2001). Drug resistance in Indian visceral leishmaniasis. Trop. Med. Int. Health.

[11] Das V, Ranjan A (2005). Magnitude of unresponsiveness to sodium stibogluconate in the treatment of visceral leishmaniasis in Bihar. Nat. Med. J. India.

[12] Haouas N, Gorcii M, Chargui N, Aoun K, Bouratbine A, Messaadi A, Masmoudi M, Zili J, Ben Said M, Pratlong F, Dedet J, Mezhoud H, Azaiez R, Babba H (2007). Leishmaniasis in central and southern Tunisia: Current geographical distribution of zymodemes. Parasite.

[13] Bombardelli E, Morazzonni P, Vitisvinifera L (1995). Fitoterapia.

[14] Baytop T (1999). Bitkiler ˙Ile Tedavi (Gec¸mis¸teveBugün).

[15] Kallel K, Haouas N, Pratlong F, Kaouech E, Belhadj S, Anane S, Dedet J, Babba H, Chaker E (2008). La leishmaniose cutanée due à Leishmania infantum MON-24 en Tunisie : extension du foyer vers le centre du pays. Bull. Soc. Pathol. Exot.

[16] Kapoor L (1990). Handbook of Ayurvedic Medicinal Plants.

[17] Felicio J, Santos R, Gonc E (2001). Chemical Constituents from Vitis vinifera (Vitaceae). Sao. Paulo.

[18] Grieve A (1971). A Modern Herbal, Dover Books New York.

[19] Onstad D (1996). Whole Foods Companion: A Guide for Adventurous Cooks, Curious Shoppers & Lovers of Natural Foods.

[20] Bown D ( 2001). The Herb Society of America.

[21] VanWyke B, Wink M (2004). Medicinal Plants of the World.

[22] Kang J, Lee W, Lee C, Yoon W, Kim N, Lee H, Park H, Han S, Yun J (2011). Improvement of high-fat dietinduced obesity by a mixture of red grape extract, soy isoflavone and l-carnitine: Implications in cardiovascular and non-alcoholic fatty liver diseases. Food Chem.Toxicol.

[23] Orhan N, Aslan M, Orhan D, Ergun F, Erdem Y (2006). In-vivo assessment of antidiabetic and antioxidant activities of grapevine leaves (Vitisvinifera) in diabetic rats. J Ethnopharmacol.

[24] Hebash K, Fadel H, Soliman M (1991). Volatile components of grape leaves. J. Islam. Acad. Sc.

[25] Nilüfer Orhan, Mustafa Aslan, Didem Deliorman Orhan, Fatma Ergun, ErdemYeşilada (2006). In-vivo assessement of antidiabetic and antioxidant activities of grape vine leaves (vitisvinifera L) in diabetic rats. J. Ethnopharmacol.

[26] Mansour R, Ayed L, Hammami S, Mighri Z, Bakhrouf A, Mhenni F (2011). Propriétés tinctoriales et Activités antibactériennes d’extraits de feuilles de Vitisvinifera L.de TUNISIE. Tunisian J. Med. Plants. Nat. Prod.

[27] Jin-Ming K, Lian-Sai C, Ngoh-Khang G, Tet-Fatt C, Brouillard R (2003). Analysis and biological activities of anthocyanins, Phytho. Chimstry.

[28] Ezzili B, Habib J, Darné G, Chemli R (1997). Influence de la date de prélèvement sur les teneurs en anthocyanes et en éléments minéraux des feuilles du cépage Alicante Bouschet cultivé à El Khengue. Bull OIV.

[29] Penchev PI (2010). PhDthesis: Étude des procédés d’extraction et de purification de produits bioactifs à partir de plantes par couplage de techniques séparatives à basses et hautes pressions. Toulouse, Toulouse University.

[30] Hmamouchi M, Es-Safi N, Essassi E (1997). Oligomeric and polymericproanthocyanidins from Moroccangrapewine (Vitisvinifera) leaves. Fitoterapia.

[31] Monagas M, Gómez-Cordovés C, Bartolomé B (2012). Evolution of the phenolic content of red wines fromVitisvinifera L. during ageing in bottle. Food Chemistry.

[32] Murphy Cowan M (1999). Plant Products as Antimicrobial Agents. Clinical Microbiol Rev.

[33] KonczakI, ZhangW (2004). Anthocyanins-More Than Nature's Colours. J. Biomed. Biotec.

[34] Aviram M, Fuhrman B (2002). Wine flavonoids protect against LDL oxidation and atherosclerosis. Ann. NY. Acad. Sci.

[35] Kolodziej H, Kiderlen A (2005). Antileishmanial activity and immune modulatory effects of tannins and related compounds on Leishmania parasitised RAW 264.7 cells. Phyto. Chemistry.

[36] Lewin G, Cojean S, Gupta A, Verma S, Puri K, Loiseau P (2011). In-vitro antileishmanial properties of new flavonoids against Leishmaniadonovani. Biomedicine and Preventive Nutrion.

[37] Nour A, Khalid S, Kaiser M, Brun R, Abdalla W, Schmidt T (2010). The antiprotozoal activity of methylated flavonoids from Ageratum conyzoides L. J. Ethno. Pharmacol.

[38] Valadares G, Duarte C, Oliveira S, Chávez-Fumagalli A, Martins T, Costa E, João V, Santoro M, Régis C, Tavares A, Coelho A (2011). Leishmanicidal activity of the Agaricusblazei Murill in different Leishmania species. Parasitol. Internat.

[39] Ghazanfari T, Hassan M, Khamesipour A (2006). Enhancement of peritoneal macrophage phagocytic activity against L. major by garlic (Allium Sativum) treatment. J. Ethno. Pharmacol.

